# Meaningful experience, positive emotions, and happiness in smart hotel contexts: a study on psychological mechanisms based on positive psychology

**DOI:** 10.3389/fpsyg.2026.1897945

**Published:** 2026-07-29

**Authors:** Jing Luo, Yibing Wang

**Affiliations:** 1 College of Business, Xi’an International University, Xi’an, China; 2 School of Economics and Management, Baoji University of Arts and Sciences, Baoji, China

**Keywords:** meaningful experience, positive emotions, positive psychology, sense of happiness, sense of perceptual control, service trust, smart hotel

## Abstract

**Introduction:**

Smart hotels are reshaping the landscape of consumers’ accommodation experiences. Services such as self-check-in and smart locks are associated with changes in consumers’ psychological perceptions of lodging life. However, previous studies have often overlooked consumers’ sense of meaning, positive emotions, and subjective happiness. Based on positive psychology and service experience theory, this study explores the psychological mechanisms and associations connecting meaningful experiences with consumer happiness.

**Methods:**

A quantitative research design was employed, analyzing data from consumers who had experienced smart hotel services (N = 618). Correlation analysis, multiple regression, and path estimation were utilized to examine the relationships among meaningful experience, perceived control, service trust, positive emotions, and happiness.

**Results:**

Respondents generally held moderately positive evaluations of the smart hotel experience, with mean scores for meaningful experience, positive emotions, and happiness at 3.837, 3.822, and 3.698, respectively. Meaningful experience was significantly and positively correlated with positive emotions (r = 0.634) and happiness (r = 0.613); the correlation between positive emotion and happiness was also significant (r = 0.603, *p* < 0.001). Regression analysis indicated that meaningful experience was a significant predictor of happiness (*β* = 0.613, *R*^2^ = 0.376); when positive emotions and other mediating variables were included, the model’s explanatory power increased to *R*^2^ = 0.490. Among them, meaningful experience had the strongest predictive association with happiness (*β* = 0.284), followed by positive emotions (*β* = 0.273), service trust (*β* = 0.196), and perceived control (*β* = 0.112). Path coefficient estimates revealed an indirect relationship between meaningful experience and happiness through positive emotions (0.117). Furthermore, sequential indirect relationships were observed for “meaningful experience → perceived control → positive emotion → happiness” (0.027) and “meaningful experience → service trust → positive emotion → happiness” (0.029).

**Discussion:**

From the perspective of positive psychology, smart hotel features are associated with a sequential pattern of psychological responses related to consumer happiness. The findings support research on the psychological mechanisms connecting meaningful experiences with happiness and offer insights for service design in the hospitality industry.

## 1 Introduction

Digital technology is profoundly transforming hotel service scenarios. Recent hospitality research indicates that artificial intelligence, service robots, smart room systems, contactless technologies, and data-driven personalization are increasingly reshaping hotel operations and guest experiences (Song et al., 2024). These technologies extend beyond operational automation by influencing consumers’ cognitive evaluations, emotional responses, service trust, and perceptions of experiential value. Services such as self-check-in, smart door locks, voice control, robot delivery, smart room environment adjustment, and personalized recommendations have shifted the hotel experience from traditional manual service to human-machine collaboration (Kim et al., 2020; Han et al., 2024). For consumers, smart hotels not only mean improved process efficiency but also a new lifestyle experience. Consumers may feel convenience, novelty, comfort, and respect in smart scenarios, but may feel uncomfortable due to complex operations, privacy concerns, or unstable services. The results show that the smart hotel experience is not simply a technical issue, but a service experience process with distinct psychological attributes. Existing smart hotel studies have mainly examined technology acceptance, perceived usefulness, service quality, customer satisfaction, and revisit intention. More recent studies have extended this stream by examining AI-enabled hospitality services, unmanned smart hotels, contactless service encounters, and service robot customer experiences. Previous research suggests that contactless services may be associated with enhanced customer experience, delight, satisfaction, and trust (Kim et al., 2021), while smart hotel technologies and service robots may shape consumers’ responses through both cognitive evaluations and affective processes (Belanche et al., 2020). Studies of unmanned smart hotels further indicate that technology-enabled services may contribute to memorable experiences and hedonic happiness when consumers perceive the servicescape, experience co-creation, and service process positively (Elshaer and Marzouk, 2024). Although prior studies help explain consumers’ willingness to adopt smart hotel technologies and whether these technologies satisfy functional expectations, they offer limited insight into how consumers interpret smart accommodation experiences as personally valuable and meaningful. In particular, relatively little attention has been given to the psychological pathways linking smart services with positive emotions and consumer happiness. Research toward smart hotels mostly focuses on technology acceptance, service quality, satisfaction, and willingness to stay again (Gursoy et al., 2019). These studies help explain whether consumers are willing to use smart hotel services, but they are still insufficient to answer a deeper question: Why does the intelligent accommodation experience enhance consumer happiness? Existing studies have generally confirmed that intelligent hotel services can improve service efficiency, convenience, and consumer satisfaction. However, the psychological consequences of smart hotel technologies remain inconsistent. Some studies suggest that self-service technologies, intelligent rooms, and personalized services may generate novelty, pleasure, and favorable service evaluations (Buhalis et al., 2019), whereas others indicate that technological complexity, privacy concerns, system instability, and reduced human interaction may cause uncertainty, anxiety, and resistance (Lu et al., 2020). These divergent findings suggest that technological convenience alone may not be sufficient to explain consumers’ happiness in smart hotel contexts.

Positive psychology research provides a possible explanation by emphasizing meaningful experience and positive emotions. Previous studies have shown that experiences perceived as valuable, purposeful, or beneficial to quality of life are often associated with greater happiness (Russo-Netzer, 2019). Positive emotions may also broaden individuals’ cognitive evaluations and facilitate favorable judgments of service experiences (Catalino and Tov, 2022). Nevertheless, previous research has not fully clarified whether meaningful experience is directly associated with happiness or whether this relationship is primarily explained by positive emotions. Moreover, meaningful technology-enabled experiences may not necessarily be associated with positive outcomes, as consumers may simultaneously encounter uncertainty, privacy concerns, and a reduced sense of control.

Perceived control and service trust may therefore represent two important psychological conditions. Consumers may recognize the value of intelligent services but still feel uncomfortable when the systems are difficult to understand or operate. Similarly, positive emotions may be weakened when consumers question system reliability, information security, or the availability of human assistance. Although previous studies have separately examined meaning, emotions, control, trust, and happiness, relatively limited research has integrated these variables into a sequential psychological mechanism in smart hotel contexts. Furthermore, emerging research on service robots shows that customer experience depends on the type and level of AI intelligence, consumers’ service goals, and the perceived performance of robotic services (Larivière et al., 2025). However, these studies have primarily focused on satisfaction, technology adoption, and usage intentions. Less attention has been paid to how AI-enabled and automated hotel services become meaningful experiences and how meaning, control, trust, and positive emotions jointly relate to consumer happiness.

Accordingly, the present study positions meaningful experience as the initial cognitive evaluation of smart hotel services, positive emotion as the central affective mechanism, and perceived control and service trust as two psychological resources. It examines whether meaningful experience predicts happiness directly and indirectly through positive emotions, as well as through the sequential paths of perceived control or service trust followed by positive emotions. This framework extends smart hotel research from technology acceptance and satisfaction to the psychological mechanisms through which intelligent accommodation experiences may be transformed into consumer happiness.

From the perspective of positive psychology, happiness does not come solely from improved external conditions but from individuals’ positive evaluations of life experiences (Berrios et al., 2018). Although positive psychology has widely examined meaning, positive emotions, perceived control, trust, and happiness, most studies have been conducted in general life, education, health, music, or traditional tourism contexts. Less is known about whether these psychological mechanisms remain applicable in highly digitalized service environments characterized by automation, human–technology interaction, and data-driven services. Smart hotels therefore provide a distinctive context in which consumers may simultaneously experience technological convenience, novelty, privacy concerns, operational pressure, and uncertainty.

Whether an individual feels life is meaningful, whether they develop positive emotions, and whether they possess a sense of control and security all affect their subjective happiness. As a new type of consumption scenario, smart hotels provide real-world scenarios for observing these positive psychological mechanisms (Koo et al., 2021). Meaningful experience is the core variable of this article. It emphasizes consumers’ comprehensive perception of service value, living convenience, future imagination, and the significance of consumption choices within smart hotels. Compared to ordinary satisfaction, meaningful experience focuses on “why this experience matters.” When consumers believe that smart hotel services improve life efficiency, enhance travel quality, and encourage positive imagination about future life, smart hotels are not just accommodation tools but may become meaningful experiential scenarios (Catalino and Tov, 2022). Positive emotions are an important psychological channel for experiencing meaning and influencing happiness. Positive psychology believes that positive emotions expand an individual’s cognitive range, making it easier for them to form open, optimistic, and constructive evaluations. When consumers experience ease, pleasure, novelty, and anticipation in smart hotels, they tend to form positive evaluations of their accommodation and further enhance their happiness (Fredrickson and Losada, 2005). At the same time, whether smart services are clear, controllable, and reliable is associated with consumers’ psychological responses. If consumers understand and control smart devices and trust the hotel system to be safe and stable, their positive emotions are easily activated and their happiness is stable. Therefore, this paper constructs a psychological mechanism model centered on smart hotel scenarios, focusing on three key questions: First, does meaningful experience positively predict consumer happiness; Second, whether positive emotions play a mediating role between meaningful experience and happiness; Third, whether perceived control and service trust further explain the psychological pathways by which Meaningful experience is associated with happiness. Through the above analysis, this study seeks to advance smart hotel research from technology adoption and satisfaction evaluation to the level of positive psychological mechanisms. Specifically, it introduces meaningful experience as a construct distinct from customer satisfaction, applies positive psychology to a highly digitalized hospitality setting, and explains how meaningful experience may be associated with happiness through positive emotions, perceived control, and service trust.

## 2 Theoretical foundations and research hypotheses

### 2.1 An integrated theoretical framework for smart hotel experience

#### 2.1.1 Technology acceptance and smart tourism ecosystem

The Technology Acceptance Model (TAM; Davis, 1989) suggests that perceived usefulness and perceived ease of use are associated with individuals’ attitudes toward and acceptance of technology. In smart hotel settings, consumers may be more willing to adopt self-check-in systems, smart room controls, facial recognition technologies, and service robots when these technologies are perceived as useful, convenient, and easy to operate. Together, these factors help explain differences in consumers’ willingness to use smart hotel services and how their evaluations of technological convenience may be associated with positive or negative psychological responses. However, smart hotel experiences are not created by a single device. Smart Tourism Ecosystem Theory (Gretzel et al., 2015) views smart tourism as an interconnected system involving consumers, hotels, digital platforms, intelligent devices, data resources, and other tourism stakeholders. From this perspective, meaningful smart hotel experiences depend on the coordination of technological functions, service processes, information systems, and human support.

#### 2.1.2 Service value and customer experience formation

Service-Dominant Logic (Vargo and Lusch, 2014) holds that value is not produced solely by service providers but is co-created through interactions among consumers, service employees, technologies, and other resources. In smart hotels, consumers are not passive recipients of intelligent services. They actively choose, operate, interpret, and evaluate smart technologies. Meaningful experience therefore emerges through value co-creation between consumers and the smart service system.

Smart hotels create experiential value beyond basic functional benefits through novelty, convenience, personalization, and aesthetic design. Accordingly, meaningful experience in smart hotels is not generated by one technological function but by consumers’ overall interpretation of self-check-in, room control, service interaction, environmental comfort, and problem-solving support.

#### 2.1.3 Positive psychology and psychological outcomes

Smart hotel experiences involve not only consumers’ psychological responses but also their acceptance of intelligent technologies, interaction with service systems, and participation in value creation. Therefore, a single theoretical perspective is insufficient to explain the formation of meaningful experiences and happiness in smart hotel contexts. This study integrates technology acceptance theories, hospitality experience theories, and positive psychology to construct a multilevel theoretical framework. Specifically, the Technology Acceptance Model explains initial technology adoption, while Smart Tourism Ecosystem Theory and Service-Dominant Logic explain how service value is co-created within an interconnected network. Positive psychology further explains how these service experiences are transformed into meaning, positive emotions, perceived control, trust, and happiness. Positive psychology emphasizes individual strengths, positive emotions, a sense of meaning, engaged experiences, and happiness. Unlike traditional problem-oriented research, positive psychology focuses on how individuals gain positive experiences in daily life and improves quality of life through meaning construction, emotional activation, and the accumulation of psychological resources (Growney et al., 2025). As a modern digital service scenario, smart hotels not only provide accommodation functions but may also become an important space for consumers to perceive technological advancement, convenience in life, and future lifestyles. In smart hotel scenarios, consumers continuously interact with intelligent systems, spatial environments, and service processes (Lin, 2022; Law et al., 2024). If smart services reduce waiting, lower uncertainty, improve comfort, and make consumers feel their lives are efficient and high-quality, consumers are likely to form positive judgments. This judgment of meaning is not merely an evaluation of technological function but a psychological identification with modern lifestyles (Lamont, 2011, 2012). Thus, the smart hotel experience can be understood as a positive service experience, where consumers gain a sense of meaning, pleasure, control, and security, ultimately forming a sense of happiness.

#### 2.1.4 Meaningful experience in smart hotel contexts

Meaningful experience refers to consumers’ subjective interpretation of the value of a service context. In smart hotels, it is not merely a preference for a particular technological feature but an overall evaluation of intelligent accommodation (Lee et al., 2014). To conceptually distinguish it from related constructs, meaningful experience goes beyond perceived usefulness, which strictly concerns whether technology improves task performance, and customer satisfaction, which reflects a post-consumption evaluation based on expectation confirmation. Furthermore, while experiential value typically assesses the trade-off between perceived benefits and costs, and memorable experiences focus on the intensity and recallability of an event, meaningful experience involves a deeper eudaimonic judgment of whether an experience is personally significant, consistent with consumers’ values and preferred lifestyles, and beneficial to their quality of life (Russo-Netzer, 2019).

In smart hotel contexts, meaningful experience may emerge when consumers interpret technological convenience as saving valuable time, enhancing autonomy, improving accommodation quality, or representing a desirable future lifestyle. Self-check-in may improve service efficiency, smart room systems may enhance comfort and control, and robotic delivery or intelligent customer service may demonstrate the potential of technology to improve accommodation experiences. Research on unmanned smart hotels also suggests that technology-enabled experiences become psychologically valuable when guests perceive the servicescape as supportive, participate in experience co-creation, and form memorable evaluations (Sthapit et al., 2024). Together, these experiences may contribute to the perception that accommodation life is valuable.

Self-determination theory explains this process through the satisfaction of autonomy, competence, and relatedness needs. Smart hotel services may support autonomy by allowing consumers to select and adjust services independently, enhance competence when intelligent systems are understandable and manageable, and maintain relatedness through personalized services and timely human support. When these needs are supported, consumers are more likely to interpret intelligent services as personally valuable rather than merely technologically efficient.

Positive psychology further distinguishes temporary pleasure from eudaimonic happiness. Convenience and novelty may generate immediate enjoyment, whereas eudaimonic happiness depends on whether an experience is meaningful and consistent with an individual’s values and preferred way of life. Thus, when consumers perceive that smart hotel services support personal choice, improve travel quality, and contribute to a desirable lifestyle, meaningful experience may connect specific service encounters with a broader and more enduring sense of happiness.

Based on this, hypotheses are proposed:

*H1*: Meaningful experience in smart hotel scenarios positively predict consumer happiness.

### 2.2 The mediating role of positive emotions

Positive emotions refer to consumers’ situation-specific affective responses during or immediately after smart hotel experiences, including pleasure, relaxation, interest, excitement, and anticipation. Unlike meaningful experience, which reflects consumers’ cognitive interpretation of experiential value, positive emotions emphasize how consumers feel during service interactions. They also differ from happiness, which represents a broader and relatively stable evaluation of the overall experience.

Positive psychology suggests that positive emotions can broaden individuals’ cognitive and behavioral resources, encouraging openness and engagement with new experiences. In AI-enabled hospitality encounters, these emotions may be shaped by consumers’ evaluations of technological performance, intelligence, social qualities, complexity, and risk. Favorable service robot performance may create excitement and enhance hotel satisfaction, while advanced AI capabilities may enrich the service robot customer experience. Individuals in positive emotional states may also be more willing to explore new services and form favorable experiential evaluations (Liu and Sun, 2025).

Smart hotel services are innovative and interactive, but technological novelty alone may not consistently generate positive emotions. When consumers interpret intelligent services as valuable and meaningful, they are more likely to experience pleasure, relaxation, and anticipation (Mongrain et al., 2016). Hospitality service robot research similarly indicates that consumers’ responses result from the interaction between cognitive evaluations and affective reactions.

Accordingly, meaningful experience, positive emotions, and happiness represent related but distinct constructs. Meaningful experience reflects cognitive value interpretation, positive emotions represent situational affective responses, and happiness reflects an integrated positive evaluation. The proposed mediation mechanism therefore suggests that consumers first interpret smart hotel services as meaningful, subsequently develop positive emotions, and then form a broader evaluation of happiness.

Based on this, a hypothesis is proposed:

*H2a*: Meaningful experience positively predicts positive emotions.

*H2b*: Positive emotions positively predict consumer happiness.

*H2c*: Positive emotions mediate the relationship between meaningful experience and happiness.

### 2.3 The chain effect of perceived control sense

Perceived control refers to the degree to which consumers believe they understand, choose, and control smart hotel services. The importance of control is particularly evident in smart hotel settings, where consumers are required to interact with automated check-in systems, intelligent rooms, mobile applications, and service robots. Recent smart hotel research indicates that perceived behavioral control, technology readiness, and consumers’ confidence in managing intelligent products are important components of smart product adoption. When automated services are understandable and manageable, consumers are more likely to form favorable emotional and behavioral responses.

Theoretically, while a foundational sense of control might act as an antecedent for initial technology adoption, this study focuses on the psychological process based on Cognitive Appraisal Theory (Lazarus, 1991). When consumers appraise the smart hotel environment as inherently meaningful and aligned with their lifestyle goals (primary appraisal), this deep sense of purpose drives cognitive engagement and psychological empowerment (Park, 2010). Consequently, this meaning-making process sequentially enhances their subjective perceived control over the environment. Positive psychology emphasizes that an individual’s sense of autonomy and control is an important psychological resource for happiness. When individuals believe they have made active choices, effectively coped with, and controlled their environment, they tend to develop positive emotions and a sense of security. Conversely, when individuals feel unable to understand or control their environment, even if external services are convenient, anxiety and rejection may arise (Nawijn and Biran, 2019).

While smart services improve convenience, they may bring operational pressure. If consumers cannot understand device functions or feel they cannot solve usage issues, the smart hotel experience may actually diminish positive feelings. When smart hotel service design is clear, consumers can independently choose whether to use smart devices and receive timely support when problems arise, enhancing their sense of control. A high sense of control reduces technological anxiety, making it easier for consumers to develop a relaxed, pleasant, and safe positive mood. Thus, Meaningful experience may further stimulate positive emotions by enhancing a sense of perceptual control, ultimately boosting happiness.

Based on this, a hypothesis is proposed:

*H3a*: Meaningful experience positively predicts perceived control.

*H3b*: Perceived control positively predicts consumer happiness.

*H3c*: Perceived control and positive emotions sequentially mediate the relationship between meaningful experience and happiness.

### 2.4 The chain effect of service trust

Service trust refers to the degree of consumer confidence in the reliability, information security, and service stability of smart hotel systems. From a positive psychology perspective, trust provides individuals with psychological security (Nelson-Coffey et al., 2023). Only when individuals believe the environment is reliable, relationships are stable, and risks are controllable can positive emotions and happiness easily form. Smart hotels rely heavily on data collection, intelligent recognition, and personalized recommendations; if consumers worry about privacy leaks, system failures, or service failures, their positive emotions are affected.

Regarding the causal sequence, while basic system trust can act as a precondition for initial technology adoption, service trust in a highly interactive smart ecosystem is continuously updated through cognitive evaluation. According to the halo effect and value congruence literature, when individuals perceive a service encounter as deeply meaningful and valuable to their well-being, this robust psychological identification generates a positive cognitive bias toward the service provider’s benevolence and integrity. Consequently, recognizing the profound meaning of smart hotel services actively reinforces consumers’ confidence and trust in the system’s reliability. Service trust may subsequently facilitate the transformation of meaningful technological experiences into positive emotions because consumers are more likely to enjoy smart services when the system is perceived as reliable, understandable, and low in risk.

Evidence from contactless hospitality services shows that a pleasant technology-mediated experience can strengthen customer equity, delight, satisfaction, and trust (Hao and Chon, 2022). Trust is therefore not merely a general psychological state but a context-specific evaluation of whether automated and contactless hotel services are reliable, secure, and capable of delivering the expected service. Consumers not only need to feel that smart hotels “make sense,” but also believe that this meaning is built on safe and reliable service. When consumers trust that smart hotels protect their personal information, operate stably, and promptly address issues, their psychological sense of security increases, and they may relax to enjoy smart services, deriving pleasure and happiness from them (Rahmani et al., 2019). Similarly, consumers’ acceptance of service robots is shaped by cognitive evaluations and affective responses to technological reliability, usefulness, and risk (Huang et al., 2024). When smart services are perceived as dependable and secure, consumers may experience lower uncertainty and stronger positive emotions.

Based on this, hypotheses are proposed:

*H4a*: Meaningful experience positively predicts service trust.

*H4b*: Service trust positively predicts consumer happiness.

*H4c*: Service trust and positive emotions sequentially mediate the relationship between meaningful experience and happiness.

## 3 Research methods

### 3.1 Samples and data sources

This study uses a questionnaire survey method to collect data, targeting consumers aged 18 and above, with a primary focus on their perceptions of smart hotel check-ins or related smart service experiences. The survey was conducted from March to April 2025. The questionnaire is mainly distributed through online questionnaire platforms and is disseminated through hotel consumer communities, tourism service exchange groups, smart tourism-related discussion groups, and social media channels.

This study used a non-probability quota sampling method. Quotas were set by gender, age, and smart hotel experience to include diverse consumers (Rivera et al., 2020), aiming to avoid overrepresentation rather than achieve strict population proportions. Within each stratum, respondents were recruited through online platforms, social media, consumer communities, and referrals until target numbers were reached. The questionnaire introduction explained the study purpose, consent, voluntariness, anonymity, and confidentiality and required participants to answer based on their actual hotel stay experiences rather than mere conceptual knowledge. Screening excluded individuals under 18. All items used a five-point Likert scale, covering meaningful experience, emotions, control, trust, happiness, and behavioral intention. Invalid responses (e.g., underage, incomplete, too fast, repetitive, or inconsistent) were removed. Although quotas improved group coverage, sampling remained convenience-based rather than random. Thus, results should be interpreted cautiously when generalizing to the broader smart hotel consumer population.

A total of 820 questionnaires were distributed in this study, 789 were collected, with a response rate of 96.22%. After excluding 33 questionnaires with short response times, regular answers, missing core items, obvious contradictions in previous and subsequent answers, or those not meeting screening criteria, 756 valid questionnaires were initially obtained. To ensure measurement validity, individuals who lacked actual smart hotel experience were further excluded, resulting in a final main sample of 618 valid responses. The sample size meets the needs of subsequent correlation analysis, regression analysis, and mediation effect tests.

### 3.2 Description of population characteristics

To further explain the sample sources and structural characteristics, this paper analyzes the gender, age, education level, occupation, smart hotel experience, and hotel stay frequency of 618 respondents over the past year. These results reflect whether the sample meets the basic requirements of smart hotel consumption scenario research, providing a basis for subsequent analysis of different consumers’ psychological responses in meaningful experience, positive emotions, and happiness.

From Table 1, it can be seen that the gender distribution of the study sample was balanced, with 307 males (accounting for 49.68%); 311 were female, accounting for 50.32%, indicating no significant gender bias in the sample, which helps reduce the impact of gender structure differences on research results. In terms of age distribution, the 26–35 and 36–45 age groups accounted for a high proportion, totaling 57.93%, indicating that the sample consists of adult consumers with stable spending power and high frequency of digital service exposure. This group usually has multiple hotel stay experiences and is easily exposed to features such as self-check-in, smart rooms, and online services in smart hotels, so they highly evaluate the psychological experience in smart hotel scenarios. In terms of education level, 62.29% of respondents had a bachelor’s degree or above, indicating that the sample overall had good questionnaire understanding and a foundation in intelligent service cognition. In terms of occupational distribution, enterprise employees, government agencies/public institution personnel, and self-employed/freelancers make up a high proportion, indicating that the sample mainly comes from people with practical experience in travel, accommodation, and consumption decisions. Regarding smart hotel experience, 100% of the respondents in the final sample have actually stayed or had related experiences, which perfectly aligns with the experiential requirements of this study. This indicates a phenomenal degree of match between the sample and the research topic, providing a reliable data basis for subsequent analysis of the relationship between meaningful experience, positive emotions, and happiness. Among them, the smart hotel service most frequently accessed by respondents is self-check-in/check-out, accounting for 20.55%; Next are facial recognition or smart locks, accounting for 17.48%. This indicates that self-service, contactless, and identity recognition services have become important gateways for consumers to access smart hotels. Smart guest room environment adjustment, online reservations, and smart customer service and voice control have high contact rates, at 14.56, 14.89, and 13.75% respectively, indicating that the smart hotel experience has extended beyond the front desk to the interior of guest rooms. Consumers are not only concerned about check-in efficiency but are also beginning to focus on the improvement of comfort and convenience brought by smart devices. The exposure rates for personalized recommendation services, robot delivery, and other services were relatively low, at 7.44, 9.39, and 1.94% respectively, indicating that these services already have a certain exposure base, but there is still room for improvement in high-frequency usage in the sample. Overall, respondents have sufficient awareness and experience with typical functions of smart hotels, providing a practical basis for evaluating meaningful experiences, positive emotions, perceived control, service trust, and happiness. At the same time, differences in service exposure ratios suggest that smart hotel consumers’ positive experiences often come from high-frequency, low-threshold, and easily perceptible service segments, such as self-check-in, facial recognition, smart door locks, and intelligent room adjustments.

**Table 1:** Population characteristic variables (*N* = 618).

Variable	Category	Number of people	Percentage (%)
Gender	Male	307	49.68
	Female	311	50.32
Age	18–25	109	17.64
	26–35	191	30.91
	36–45	167	27.02
	46–55	101	16.34
	56 years old and above	50	8.09
Education level	High school and below	80	12.94
	Junior college	153	24.76
	Bachelor’s degree	283	45.79
	Master’s degree or above	102	16.50
Occupation	Student	51	8.25
	Corporate employees	219	35.44
	Staff of government agencies/public institutions	115	18.61
	Enterprise managers	74	11.97
	Self-employed/freelancer	112	18.12
	Others	47	7.61
Smart hotel experience	Yes	618	100.00
Hotel stay frequency over the past year	Once or fewer	115	18.61
	2–3 times	203	32.85
	4–6 times	144	23.30
	7–10 times	99	16.02
	11 times or more	57	9.22
Annual income	Below 30,000 yuan	81	13.11
	30,000–60,000 yuan	189	30.58
	60,000–100,000 yuan	193	31.23
	100,000–150,000 yuan	109	17.64
	150,000 yuan or more	46	7.44
The smart hotel services you have encountered	Online appointment and intelligent customer service	92	14.89
	Self check-in/check-out	127	20.55
	Smart guest room environment adjustment	90	14.56
	Intelligent voice control	85	13.75
	Facial recognition or smart door locks	108	17.48
	Personalized recommendation services	46	7.44
	Robot delivery	58	9.39
	Others	12	1.94

### 3.3 Questionnaire quality control

To ensure the quality of questionnaire data, this study established quality control procedures during the design, distribution, and collection stages of the questionnaire. Before the official distribution of the questionnaire, the study conducted preliminary tests on some consumers with hotel stay experience and experience with smart hotel services, and adjusted the wording of the items, the order of the questionnaire, and instructions based on feedback to ensure the questions were clear in meaning and expressed naturally. During formal surveys, questionnaires are completed anonymously, emphasizing that there is no right or wrong answer, and all data are used solely for academic research to reduce social bias and defensive responses.

During the formal survey, the questionnaire included age screening questions and smart hotel cognition screening questions. For respondents under 18, the system automatically terminates the response. Respondents who had no hotel stay experience and were completely unfamiliar with smart hotel services were excluded from the valid sample range. After data collection, questionnaires with obviously short completion times, missing core items, consecutive selection of the same option, demographic information contradictions, and non-compliance with screening criteria were further removed. After the above screening, and the rigorous exclusion of participants without actual smart hotel experience, 618 valid questionnaires were retained.

### 3.4 Questionnaire validity analysis

To ensure the questionnaire accurately measures meaningful experience, positive emotions, perceived control, service trust, and happiness in smart hotel scenarios, it is necessary to first determine whether the questionnaire items are suitable for factor analysis and whether each item effectively reflects their respective variables. Therefore, this paper used KMO test, Bartlett sphericity test, and exploratory factor analysis, and tested the structural validity of the questionnaire. The specific results are shown in Table 2.

**Table 2:** Results of the questionnaire structural validity test.

Inspection indicators	The result	Judgment criteria	Conclusion
KMO value	0.956	>0.800	Suitable factor analysis
Bartlett Spherical inspection χ^2^	7119.168	*p* < 0.001	Reaching a significant level
Degrees of freedom *df*	528	—	—
Five factors cumulatively explain variance	52.091%	>50%	Acceptable
Minimum factor loading	0.552	>0.500	The load for the item is acceptable
Maximum factor loading	0.696	>0.500	The item load is good

Table 2 shows that the KMO value of the questionnaire in this study is 0.956, significantly higher than the criterion of 0.800 (Hair Jr et al., 2010), indicating strong correlations among variables, and the sample data are very suitable for factor analysis. Bartlett’s spherical test results reached a significant level, *χ*^2^ = 7119.168, *p* < 0.001, further indicating that the items are not independent of each other but share a common factor structure, allowing subsequent structural validity tests.

From the perspective of factor explanation, the cumulative variance of the five factors was 52.091%, higher than the 50% basic criterion (Kaiser, 1974), indicating that the five dimensions of meaningful experience, positive emotions, perceived control, service trust, and happiness explained most of the questionnaire information. The overall factor load for each item is above 0.50, with a minimum load of 0.552, with a maximum load of 0.696, indicating that each item has a good correspondence with its corresponding dimension. Overall, the questionnaire’s structural validity was at an acceptable level and could be used for subsequent correlation analysis, regression analysis, and path estimation. After structural validity testing, this paper further examines whether the internal items of each variable are stably aggregated into the same latent concept. Mean variance extraction and combinatorial reliability can reflect the explanatory ability and internal consistency of scale items for latent variables. Therefore, this paper conducts aggregation validity tests on five core variables, with the following results.

As shown in Table 3, the factor loadings of each measurement item on its corresponding construct are clearly higher than their maximum cross-loadings on other constructs. Specifically, the factor loadings of Meaningful Experience range from 0.587 to 0.683, with a maximum cross-loading of approximately 0.284; Positive Emotions range from 0.552 to 0.654, with a maximum cross-loading of approximately 0.249; Perceived Control ranges from 0.644 to 0.694, with a maximum cross-loading of approximately 0.194; Service Trust ranges from 0.652 to 0.696, with a maximum cross-loading of approximately 0.229; and happiness ranges from 0.599 to 0.690, with a maximum cross-loading of approximately 0.266. For all constructs, the minimum factor loading is higher than the corresponding maximum cross-loading, indicating that each item effectively reflects its intended construct and demonstrates good discriminant validity from other constructs. Therefore, the cross-loading results support that the measurement model has satisfactory discriminant validity. To further evaluate whether the proposed five-factor measurement structure adequately fits the observed data, confirmatory factor analysis was conducted. Multiple goodness-of-fit indices were examined, including *χ*^2^/*df*, GFI, AGFI, CFI, NNFI, TLI, IFI, RMSEA, SRMR, and RMR. The results are presented in Table 4.

**Table 3:** Cross-loadings test of discriminant validity results.

Construct items	Construct	Loading range	Maximum cross-loading	Judgment
X1–X7	Meaningful Experience (X)	0.587–0.683	0.284	Pass
PE1–PE7	Positive emotions (PE)	0.552–0.654	0.249	Pass
PC1–PC6	Perceptual Control (PC)	0.644–0.694	0.194	Pass
ST1–ST6	Service Trust (ST)	0.652–0.696	0.229	Pass
Y1–Y7	Happiness(Y)	0.599–0.690	0.266	Pass

**Table 4:** Confirmatory factor analysis (CFA) model fit indices (*N* = 618).

Index	Value	Recommended Threshold	Result
χ^2^	504.580	—	—
df	485	—	—
χ^2^/df	1.040	<3	Excellent
GFI	0.931	>0.90	Excellent
AGFI	0.924	>0.90	Excellent
CFI	0.997	>0.90	Excellent
NFI	0.931	>0.90	Good
NNFI (TLI)	0.997	>0.90	Excellent
IFI	0.997	>0.90	Excellent
RMSEA	0.008	<0.08	Excellent
SRMR	0.025	<0.08	Excellent
RMR	0.019	<0.05	Excellent

As shown in Table 4, the confirmatory factor analysis results indicate that the proposed measurement model demonstrates an excellent overall fit. The χ^2^/df value was 1.040, which was below the recommended threshold of 3 (Kline, 2023). The GFI and AGFI values were 0.931 and 0.924, respectively, both exceeding 0.90. In addition, the CFI, TLI, and IFI values were all 0.997, while the NFI value was 0.931, indicating satisfactory incremental fit (Hu and Bentler, 1999). The RMSEA value was 0.008, with SRMR and RMR values of 0.025 and 0.019, respectively, all below the recommended thresholds (Hair Jr et al., 2010). Overall, these results suggest that the five-factor measurement model fits the observed data well and provides support for the proposed construct structure.

### 3.5 Common method deviation testing

Since the data in this study were collected through a self-reported questionnaire survey, procedural remedies were applied to reduce potential common method variance. Specifically, the questionnaire was administered anonymously, and respondents were informed that there were no right or wrong answers and that the data would be used solely for academic research purposes.

At the statistical level, Harman’s one-factor test was conducted to assess the potential presence of common method bias. The results of the unrotated exploratory factor analysis indicate that the first factor accounts for 31.84% of the total variance, which is below the commonly used threshold of 40%. This suggests that no single factor dominates the explanation of the variance structure. Overall, the results indicate that common method variance is unlikely to be a serious concern in this study. The results show that the observed relationships among variables are more likely to reflect their underlying associations rather than being driven primarily by measurement method effects. Accordingly, the data are considered appropriate for further statistical analysis.

As shown in Table 5, the single-factor model demonstrates poor model fit, with low CFI (0.697) and high RMSEA (0.119) and SRMR (0.103), indicating that a single latent factor does not adequately account for the observed covariance among variables. In contrast, the five-factor measurement model exhibits excellent fit indices (CFI = 0.997, TLI = 0.997, RMSEA = 0.008, SRMR = 0.025), suggesting that the proposed multi-construct structure provides a substantially better representation of the data. When an unmeasured latent method factor was introduced, the model fit improved only marginally compared with the five-factor model (changes in key fit indices were less than 0.01). Overall, these results suggest that common method variance is unlikely to be a major concern in this study. However, given that all data were collected from the same respondents using self-reported questionnaires at a single time point, potential method bias cannot be completely ruled out. Therefore, both the Harman single-factor test and the latent method factor approach should be interpreted as diagnostic rather than definitive evidence regarding common method bias. Additionally, the measurement model was further evaluated using standardized factor loadings, composite reliability (CR), and average variance extracted (AVE), which confirm acceptable reliability and convergent validity of the constructs.

**Table 5:** CFA test for common method bias (*N* = 618).

Model	χ^2^/df	CFI	TLI	GFI	RMSEA	SRMR	Result
Single-factor model	5.862	0.697	0.678	0.671	0.119	0.103	Poor
Five-factor model	1.040	0.997	0.997	0.931	0.008	0.025	Excellent
Method-factor model	1.025	0.998	0.998	0.942	0.006	0.022	No significant CMB

Table 6 shows that the composite reliability (CR) values of all constructs exceeded 0.80, indicating satisfactory internal consistency among the measurement items. This suggests that the indicators within each construct share an acceptable level of internal coherence in measuring the corresponding latent variables.

**Table 6:** Results of convergent validity testing.

Variable	Standardized load range	AVE	CR	Judgment results
Meaningful Experience (X)	0.698–0.748	0.520	0.846	Excellent internal consistency
Positive emotions (PE)	0.657–0.704	0.466	0.809	Acceptable
Perceptual Control (PC)	0.708–0.723	0.509	0.807	Excellent internal consistency
Service Trust (ST)	0.693–0.748	0.520	0.815	Excellent internal consistency
Happiness (Y)	0.699–0.727	0.502	0.834	Excellent internal consistency

CR > 0.70 indicates adequate internal consistency. According to Fornell and Larcker (1981), convergent validity is still acceptable if AVE is slightly below 0.50 but CR exceeds 0.60.

Specifically, the standardized factor loadings of all measurement items ranged from 0.657 to 0.748, demonstrating that each indicator loaded strongly and significantly (*p* < 0.001) on its respective latent construct.

Notably, after adhering strictly to the experiential screening criteria, the average variance extracted (AVE) values for Meaningful Experience (0.520), Perceived Control (0.509), Service Trust (0.520), and happiness (0.502) all surpassed the conservative threshold of 0.50, demonstrating solid convergent validity. Although the AVE for Positive Emotions (0.466) is slightly below 0.50, its high composite reliability (CR = 0.809) ensures that the measurement model’s convergent validity remains adequate (Fornell and Larcker, 1981).

Among the five constructs, meaningful experience and service trust showed the highest AVE (0.520), while positive emotions showed the lowest (0.466). Overall, these results indicate that the measurement model exhibits acceptable reliability and adequate convergent validity, with only a slight acceptable variance in the emotional dimension.

In summary, the measurement instrument in this study achieved satisfactory levels of content validity, structural validity, and convergent validity, providing an appropriate data foundation for subsequent correlation, regression, and mediation analyses.

### 3.6 Measurement development and variable operationalization

Happiness was measured using seven items that assessed consumers’ overall positive evaluation of the smart hotel experience, including whether the experience enhanced their sense of happiness, improved their perceived quality of accommodation life, and contributed to a positive evaluation of their experiential state. Happiness was treated as a broader outcome variable rather than an immediate emotional response. Therefore, it differs from positive emotions in duration and evaluative scope: positive emotions describe temporary feelings during the service encounter, whereas happiness reflects an integrated judgment of the overall positive value of the experience.

The questionnaire was designed to assess consumers’ psychological evaluations of smart hotel services. It included five core constructs: meaningful experience, positive emotions, perceived control, service trust, and happiness. All items were measured using a five-point Likert scale ranging from 1 (“strongly disagree”) to 5 (“strongly agree”), with higher scores indicating higher levels of the corresponding construct.

The measurement items were developed and contextually adapted with reference to the conceptual definitions and relevant literature discussed in the theoretical section. Rather than being reproduced verbatim from a single existing instrument, the items were formulated to reflect consumers’ perceptions and emotional responses in smart hotel contexts. The questionnaire referred to typical smart hotel services, including self-check-in and check-out, facial recognition or smart door locks, smart room controls, intelligent voice-control systems, online reservations, intelligent customer service, personalized recommendation services, and robotic delivery.

Meaningful experience was measured using seven items coded X1–X7. These items focused on whether consumers perceived smart hotel services as valuable, beneficial to accommodation quality, representative of technological improvements in everyday life, and consistent with their expectations of a desirable future lifestyle. Positive emotions were measured using seven items coded PE1–PE7, covering emotional responses such as pleasure, relaxation, interest, novelty, excitement, and anticipation.

Perceived control was measured using six items coded PC1–PC6. These items assessed whether consumers understood, selected, and managed smart hotel services and whether they felt capable of dealing with problems during service use. Service trust was measured using six items coded ST1–ST6, focusing on consumers’ evaluations of system reliability, service stability, privacy protection, information security, and service dependability. Happiness was measured using seven items coded Y1–Y7, reflecting respondents’ overall positive evaluation of their smart hotel experience, perceived accommodation quality, convenience, comfort, and experiential happiness.

Before the formal survey, the preliminary questionnaire was tested among consumers with hotel accommodation experience and familiarity with smart hotel services. Based on respondent feedback, the wording of selected items, the order of the questionnaire, and the survey instructions were adjusted to improve clarity, readability, and contextual relevance. The formal questionnaire was administered in Chinese. Because the current study did not employ a documented independent back-translation procedure during data collection, no formal back-translation process is claimed. The English descriptions reported in the manuscript are used to present the constructs and measurement content to international readers.

The item codes and measurement focus of each construct are summarized in Table 7. The complete wording of the questionnaire items should be provided in Supplementary material based on the original survey instrument.

**Table 7:** Measurement constructs, item codes, and development basis.

Construct	Item codes	Number of items	Measurement focus	Development basis
Meaningful experience	X1–X7	7	Personal value, technological value, accommodation quality, life improvement, and desirable future lifestyle	Developed and contextually adapted with reference to meaningful experience, experiential value, and positive psychology literature (Russo-Netzer, 2019)
Positive emotions	PE1–PE7	7	Pleasure, relaxation, interest, novelty, excitement, and anticipation	Developed with reference to positive affect and emotional responses in tourism and technology-enabled service contexts (Hosany et al., 2015)
Perceived control	PC1–PC6	6	Understanding, choice, independent operation, manageability, and problem-solving confidence	Contextually adapted with reference to perceived behavioral control, autonomy, and smart technology-use research (Ajzen, 1991)
Service trust	ST1–ST6	6	Reliability, stability, privacy protection, information security, and dependability	Contextually adapted with reference to trust in technology-enabled and contactless services (Hao and Chon, 2022)
Happiness	Y1–Y7	7	Overall positive evaluation, accommodation convenience, comfort, quality of experience, and experiential happiness	Developed with reference to subjective well-being, happiness, and positive experiential evaluation literature (Diener et al., 1985)

All items were assessed using a five-point Likert scale ranging from 1 = strongly disagree to 5 = strongly agree.

### 3.7 Data analysis methods

This study used descriptive statistics, reliability testing, validity testing, correlation analysis, regression analysis, and path coefficient estimation. First, descriptive statistical analysis was conducted on the demographic characteristics and main variables of the sample. Second, Cronbach’s *α* coefficients were used to test internal consistency of various scales, and the validity of questionnaires was assessed using KMO tests, Bartlett spherical tests, exploratory factor analysis, AVE, and CR tests. Third, Pearson correlation analysis examines the relationships among Meaningful experience, positive emotions, perceived control, service trust, and happiness. Finally, regression analysis was used to examine the pathways of action among Meaningful experience, positive emotions, perceived control, service trust, and happiness. The mediation effects were tested using the bootstrap method with 5,000 resamples. Total, direct, and indirect effects were estimated with bias-corrected 95% confidence intervals. Mediation was considered significant when the confidence interval did not include zero. During the model testing, this study controlled for gender, age, education level, annual income, and hotel stay frequency in the past year to reduce the impact of demographic variables and differences in consumption experience on the research results. Given that the sample was explicitly restricted to consumers with actual smart hotel experience, this specific parameter remained constant and did not require additional statistical control. It should be noted that the mediating effects section of this paper mainly estimates indirect effects based on regression path coefficients, primarily using PROCESS, AMOS, and SPSS 19.0 for Bootstrap repeat sampling tests, and reporting Boot SE and 95% confidence intervals.

## 4 Research results

### 4.1 Descriptive statistics and reliability testing

After confirming the basic validity of the questionnaire, this paper further conducts descriptive statistics and reliability tests for each core variable. Mean and standard deviation reflect respondents’ overall evaluation of each variable, while Cronbach’s *α* coefficient is used to assess internal consistency across scales. The specific results are as follows.

Table 8 shows that the mean scores of all core variables were well above 3.00, suggesting that respondents generally held moderately positive evaluations of smart hotel experiences. Meaningful experience had the highest mean score (M = 3.837), followed closely by positive emotions (M = 3.822). The similarity of these scores suggests that higher evaluations of the meaningfulness of smart hotel services align with relatively stronger positive emotional responses. The mean score for happiness was 3.698, indicating that smart hotel experiences were generally positively associated with respondents’ evaluations of accommodation convenience, comfort, and quality of life.

**Table 8:** Variable description statistics and reliability tests (*N* = 618).

variable	Number of items	Mean	Standard deviation	Minimum value	Maximum value	Cronbach’s α
Meaningful experience (X)	7	3.837	0.619	2.000	5.000	0.846
Positive emotions (PE)	7	3.822	0.566	1.857	5.000	0.809
Perceptual control (PC)	6	3.508	0.619	1.500	5.000	0.807
Service trust (ST)	6	3.488	0.638	1.333	5.000	0.815
Happiness (Y)	7	3.698	0.609	1.286	4.857	0.834
Overall	33	3.684	0.490	2.061	4.857	0.941

By comparison, perceived control and service trust had lower mean scores of 3.508 and 3.488, respectively. This pattern suggests that, although respondents generally reported positive evaluations of smart hotels, they appeared to retain some reservations regarding their control over intelligent services and the ultimate reliability of smart systems.

Regarding reliability, Cronbach’s *α* values for all variables exceeded the 0.80 threshold, indicating satisfactory internal consistency and support the use of these measures in the subsequent statistical analyses.

### 4.2 Correlation analysis

To preliminarily explore whether there is a statistical association among Meaningful experience, positive emotions, perceived control, service trust, and happiness, this paper uses Pearson correlation analysis to test the core variables. While correlation analysis provides the basis for relational structures, subsequent pathway analyses are conducted to estimate predictive directions. Specific results are shown in Table 9.

**Table 9:** Correlation analysis of core variables (*N* = 618).

variable	X	PE	PC	ST	Y
Meaningful Experience (X)	**0.721**				
Positive emotions (PE)	0.634***	**0.683**			
Perceptual Control (PC)	0.542***	0.487***	**0.713**		
Service Trust (ST)	0.488***	0.486***	0.330***	**0.721**	
Happiness(Y)	0.613***	0.603***	0.464***	0.505***	**0.709**

***p < 0.001. Values on the diagonal (in bold) represent the square roots of the Average Variance Extracted (AVE) for each construct, while the off-diagonal values represent the Pearson correlation coefficients between constructs.

To ensure that the constructs are empirically distinct, discriminant validity was assessed using the heterotrait–monotrait ratio (HTMT). This method is considered more robust than traditional criteria such as Fornell–Larcker. Table 9 shows that meaningful experience was significantly and positively correlated with positive emotions, perceived control, service trust, and happiness. This suggests that higher levels of perceived meaningfulness in smart hotel services were associated with stronger positive emotions, greater perceived control, higher service trust, and greater happiness. Among these relationships, the correlation between meaningful experience and positive emotions was the strongest (*r* = 0.634), indicating that these two variables were particularly closely associated. This pattern is consistent with the perspective of positive psychology, which proposes that experiences perceived as valuable and meaningful tend to be positively related to emotions such as joy, relaxation, and anticipation.

Positive emotions were also significantly positively correlated with happiness (*r* = 0.603), while meaningful experience showed a similarly strong positive correlation with happiness (*r* = 0.613). These results suggest that both meaningful experience and positive emotions may represent important psychological correlates of happiness in smart hotel contexts. In addition, service trust was positively correlated with happiness (*r* = 0.505), and this association was stronger than that between perceived control and happiness (*r* = 0.464). This finding suggests that consumers’ perceptions of system stability, privacy protection, and service reliability may be more closely associated with happiness than their perceived control over smart hotel services.

As shown in Table 10, the HTMT values among all constructs range from 0.407 to 0.767, which are well below the commonly recommended threshold of 0.85 and the more lenient threshold of 0.90. Among them, the HTMT value between meaningful experience and positive emotions is the highest at 0.767, followed by positive emotions and happiness at 0.733, and meaningful experience and happiness at 0.729. Although relatively strong correlations exist among these constructs, all HTMT values remain below the recommended thresholds for discriminant validity. The lowest HTMT value is between perceived control and service trust (0.407), indicating a clear distinction between these two constructs. Overall, the results confirm satisfactory discriminant validity among all constructs, with no evidence of construct overlap.

**Table 10:** HTMT discriminant validity test results (*N* = 618).

Variables	Meaningful experience (X)	Positive emotions (PE)	Perceptual Control (PC)	Service Trust (ST)	Happiness (Y)
Meaningful experience (X)	—				
Positive emotions (PE)	0.767	—			
Perceptual control (PC)	0.656	0.602	—		
Service trust (ST)	0.588	0.598	0.407	—	
Happiness (Y)	0.729	0.733	0.565	0.611	—

### 4.3 Collinearity test

Before conducting multiple regression analysis, this paper further tests the core predictor variables for collinearity to determine whether there is an excessive correlation among Meaningful experience, positive emotion, perceived control, and services that is associated with the stability of regression estimation. Colinearity testing mainly uses tolerance and variance inflation factors (VIF) for judgment. It is generally believed that a tolerance greater than 0.10 and a variance inflation factor less than 5 indicate that the model does not have serious multicollinearity issues.

Table 11 shows that the VIF values for Meaningful experience, positive emotion, perceived control, and service trust range from 1.411 to 2.017, all below the criterion of 5; the tolerance ranged from 0.496 to 0.709, both above 0.10. This indicates that there is no severe multicollinearity among the predictor variables, and variables can simultaneously enter the regression model. Thus, the explanatory roles of each variable in subsequent regression analyses are relatively stable, and the model estimation results have acceptable statistical reliability.

**Table 11:** Collinearity test results.

Predictor variables	Tolerance	VIF	Judgment results
Meaningful experience (X)	0.496	2.017	No severe collinearity exists
Perceptual control (PC)	0.671	1.491	No severe collinearity exists
Service trust (ST)	0.709	1.411	No severe collinearity exists
Positive emotions (PE)	0.533	1.876	No severe collinearity exists

### 4.4 Regression analysis

Although correlation analysis reveals bivariate associations, multiple regression analyses are necessary to examine the joint predictive relationships among the variables. To test the research hypotheses, this paper utilizes multiple regression analysis to examine the predictive relations of Meaningful experience, positive emotions, perceived control, and service trust on happiness, and analyzes the pathway relationships among the variables. The results are as follows.

Table 12 shows that meaningful experience was significantly associated with happiness, with a standardized regression coefficient of 0.613. The model explained 37.6% of the variance in happiness. This result suggests that higher levels of meaningful experience were associated with higher levels of happiness in smart hotel contexts. Respondents who perceived smart hotel services as more valuable, technologically meaningful, and beneficial to their accommodation experience also tended to report greater happiness. Therefore, H1 was supported. This finding suggests that the psychological value of smart hotels may be positively related not only to technological convenience but also to consumers’ subjective construction of meaning during their interactions with smart services.

**Table 12:** Regression path results (*N* = 618).

Predictor Variables	Dependent variable	Standardized *β*	*R* ^2^	Significance	Note
Meaningful experience (X)	Happiness (Y)	0.613	0.376	*p* < 0.001	Independent predictive relations of key constructs on happiness (Y).
Meaningful experience (X)	Perceptual control (PC)	0.542	0.294	*p* < 0.001	
Meaningful experience (X)	Service trust (ST)	0.488	0.238	*p* < 0.001	
Meaningful experience (X)	Positive emotions (PE)	0.634	0.402	*p* < 0.001	
Perceptual control (PC)	Positive emotions (PE)	0.487	0.237	*p* < 0.001	
Service trust (ST)	Positive emotions (PE)	0.486	0.236	*p* < 0.001	
Meaningful experience (X)	Happiness (Y)	0.284	0.490	*p* < 0.001	After adding positive emotions (PE), perceived control (PC), and service trust (ST), the association of Meaningful experience (X) on happiness (Y).
Positive emotions (PE)	Happiness (Y)	0.273		*p* < 0.001	
Perceptual control (PC)	Happiness (Y)	0.112		*p* = 0.001	
Service trust (ST)	Happiness (Y)	0.196		*p* < 0.001	

Meaningful experience was also significantly positively associated with perceived control and service trust, with standardized regression coefficients of 0.542 and 0.488, respectively. The corresponding models explained 29.4 and 23.8% of the variance in these variables. These results indicate that higher levels of meaningful experience were associated with stronger perceived control and greater service trust. Consumers who regarded their smart hotel experiences as more meaningful also tended to perceive smart services as more understandable, controllable, reliable, and trustworthy. In the positive-emotion models, meaningful experience, perceived control, and service trust were all significantly positively associated with positive emotions. The standardized coefficient for the relationship between meaningful experience and positive emotions was 0.634, and the model explained 40.2% of the variance. The standardized coefficients for perceived control and service trust were 0.487 and 0.486, with corresponding explained variance values of 23.7 and 23.6%. These findings suggest that higher levels of perceived meaning, control, and service trust were associated with stronger positive emotional responses.

In the multiple regression happiness model, meaningful experience remained significantly positively associated with happiness after positive emotions, perceived control, and service trust were included, with a standardized coefficient of 0.284. The model explained 49.0% of the variance in happiness. Positive emotions, perceived control, and service trust were also significantly positively associated with happiness, with standardized coefficients of 0.273, 0.112, and 0.196, respectively. Among these variables, positive emotions showed the strongest positive association with happiness, suggesting that they may represent a central psychological pathway linking smart hotel experiences with consumer happiness.

To examine the effects of the core independent variables and control variables on happiness, a multiple linear regression analysis was conducted. The results, including regression coefficients and multicollinearity diagnostics, are presented in Table 13.

**Table 13:** Control variables and main regression results (*N* = 618).

Variable	*B*	*SE*	Beta	*t*	*p*	95% CI	VIF
Constant	0.492	0.165	—	2.976	0.003	[0.167, 0.816]	—
X (Meaningful Experience)	0.266	0.041	0.270	6.536	<0.001***	[0.186, 0.345]	2.084
PE (Positive Emotion)	0.298	0.042	0.277	7.016	<0.001***	[0.215, 0.381]	1.898
PC (Perceived Control)	0.112	0.035	0.114	3.235	0.001**	[0.044, 0.180]	1.512
ST (Service Trust)	0.198	0.033	0.208	6.060	<0.001***	[0.134, 0.263]	1.433
Age	−0.028	0.015	−0.054	−1.822	0.069	[−0.058, 0.002]	1.091
Gender	−0.057	0.035	−0.047	−1.626	0.104	[−0.126, 0.012]	1.018
Education level	−0.012	0.020	−0.018	−0.631	0.528	[−0.051, 0.026]	1.028
Occupation	0.037	0.012	0.089	3.014	0.003**	[0.013, 0.061]	1.069
Annual income	−0.009	0.016	−0.017	−0.575	0.565	[−0.040, 0.022]	1.025
Hotel stay frequency	0.025	0.015	0.049	1.676	0.094	[−0.004, 0.053]	1.046

**p < 0.01, ***p < 0.001. Categorical demographic variables (i.e., gender, education, occupation, income, and stay frequency) were transformed using dummy coding prior to model entry. *R*^2^ = 0.503, Adjusted *R*^2^ = 0.495, *F* = 61.470, *p* < 0.001, Durbin-Watson = 2.047.

As shown in Table 13, the main explanatory variables (meaningful experience, positive emotions, perceived control, and service trust) all exhibit significant positive associations on happiness. Specifically, positive emotions show the strongest influence (*β* = 0.277, *p* < 0.001), followed by meaningful experience (*β* = 0.270, *p* < 0.001) and service trust (*β* = 0.208, *p* < 0.001). Perceived control also demonstrates a significant positive association (*β* = 0.114, *p* = 0.001).

In contrast, most control variables, including age, gender, education, income, and visit frequency, are not statistically significant, indicating that demographic characteristics do not significantly influence happiness in this model, with the exception of occupation (*β* = 0.089, *p* = 0.003). Notably, since the study target was defined exclusively as consumers with actual smart hotel stay experience, this characteristic remained constant across the sample and was omitted from the covariate controls. In addition, multicollinearity diagnostics indicate that all VIF values are below 3, suggesting that there is no serious multicollinearity problem among the variables. The model explains a substantial proportion of variance in happiness (*R*^2^ = 0.503, Adjusted *R*^2^ = 0.495), demonstrating good explanatory power.

### 4.5 Mediation path estimation

Based on the regression path results, this paper further estimates the indirect pathways predicting happiness through Meaningful experience based on standardized path coefficients. This analysis helps reveal the psychological mechanisms that positive emotions, perceived control, and service trust may play between meaningful experience and happiness. A bias-corrected bootstrapping method with 5,000 resamples was used to calculate the 95% confidence intervals (CIs) for both total, direct, and specific indirect pathways. The specific estimates are as follows.

In Table 14, the results indicate that meaningful experience is positively associated with happiness in smart hotel contexts. The total effect (*β* = 0.613) demonstrates a strong positive relationship, implying that higher meaningful experience is associated with higher happiness. After including mediators, the direct effect remains positive (*β* = 0.284), indicating that meaningful experience retains a direct positive association with happiness, although part of its predictive power operates through mediating mechanisms.

**Table 14:** Estimation of indirect effects based on bootstrapping (95% CI).

Effect type	Path	Standardized Effect	Boot SE	Boot LLCI	Boot ULCI	Result
Total effect	X → Y	0.613	0.033	0.549	0.678	Significant
Direct effect	X → Y	0.284	0.042	0.202	0.366	Significant
Total indirect	X → X → Y	0.329	0.035	0.260	0.399	Significant
Specific indirect 1	X → PE → Y	0.117	0.020	0.080	0.159	Significant
Specific indirect 2	X → PC → PE → Y	0.027	0.007	0.015	0.041	Significant
Specific indirect 3	X → ST → PE → Y	0.029	0.007	0.017	0.043	Significant
Specific indirect 4	X → PC → Y	0.061	0.019	0.025	0.097	Significant
Specific indirect 5	X → ST → Y	0.095	0.019	0.060	0.135	Significant

The total indirect effect (*β* = 0.329, 95% CI [0.260, 0.399]) excludes zero, confirming that meaningful experience predicts happiness through indirect pathways. Specifically, the total indirect effect is composed of five distinct significant pathways. Of these, the single-mediator path through positive emotions is the strongest (*β* = 0.117, 95% CI [0.080, 0.159]). The sequential mediation pathways via perceived control (*β* = 0.027, 95% CI [0.015, 0.041]) and service trust (*β* = 0.029, 95% CI [0.017, 0.043]) also show significant positive associations. Crucially, as requested by the analytical framework, the direct mediating relationships skipping the emotional stage were also estimated; the paths through perceived control (*β* = 0.061, 95% CI [0.025, 0.097]) and service trust (*β* = 0.095, 95% CI [0.060, 0.135]) are both statistically significant. The sum of these five specific indirect effects (0.117 + 0.027 + 0.029 + 0.061 + 0.095) mathematically equals the total indirect effect of 0.329. Comparatively, the service trust pathway appears slightly stronger than perceived control, indicating that system reliability and security are more closely associated with emotional responses and happiness in smart hotel contexts.

**Figure 1:**
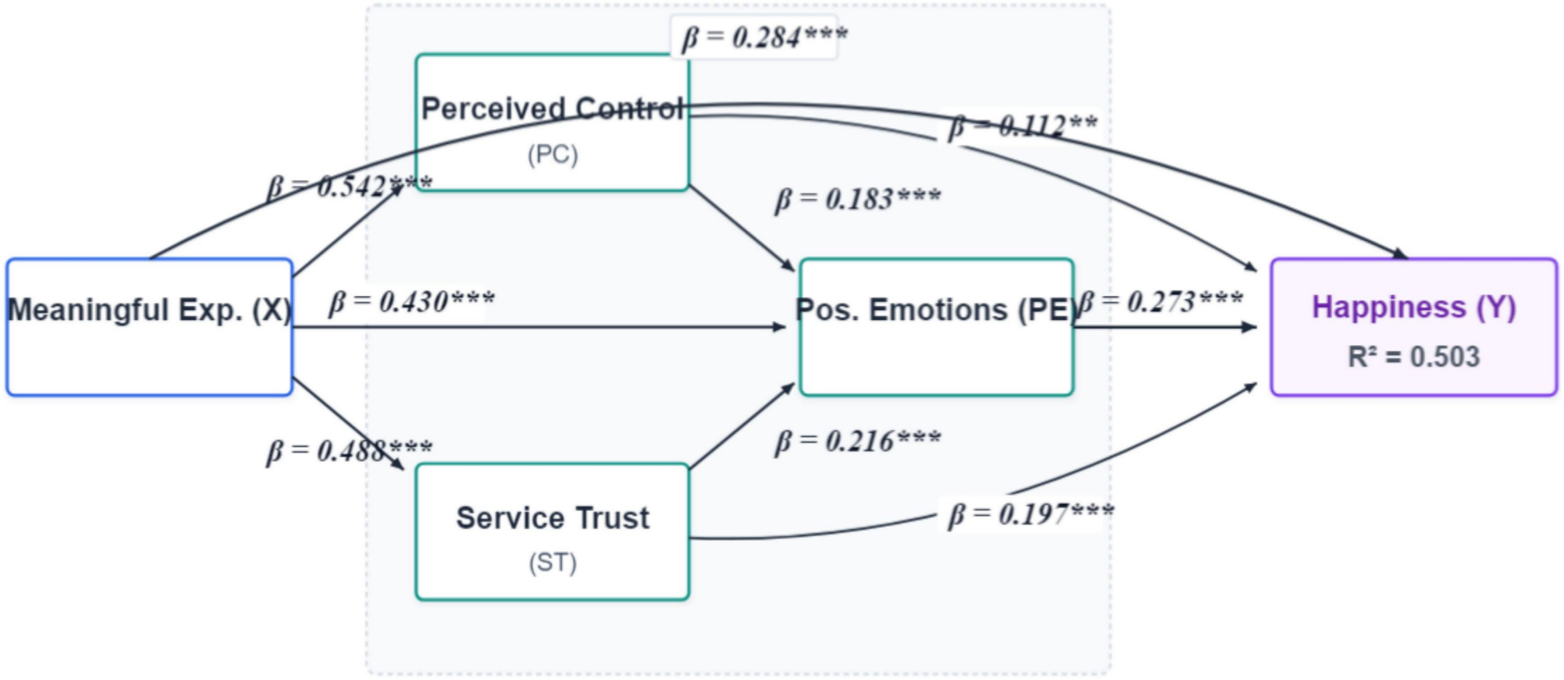
SEM path model of meaningful experiences affecting happiness.

Overall, the findings suggest that meaningful experience, positive emotions, perceived control, and service trust are positively associated with happiness, with positive emotions playing a central mediating role. The conceptual framework of the research findings is shown in Figure 1.

Figure 1 illustrates the conceptual framework of meaningful experiences affecting happiness. Meaningful experiences predict happiness directly, as well as indirectly through positive emotions, perceived control, and service trust. Among these, perceived control and service trust further are positively associated with positive emotions, forming a chain of intermediary pathways. This framework illustrates the multiple psychological pathways through which smart hotel experiences are associated with consumer happiness.

Although both sequential indirect associations had relatively small path coefficients (*β* = 0.027 and *β* = 0.029, respectively), they indicate that perceived control and service trust are positively related to the transformation of meaningful experience into positive emotional responses and happiness. Specifically, stronger meaningful experience is associated with higher perceived control and greater service trust, which subsequently relate to more positive emotions and higher happiness.

This difference suggests that system reliability, privacy protection, and service stability within the service trust pathway have a somewhat closer positive association with happiness in automated hotel settings than control mechanisms. Overall, the results provide support for the positive associations proposed in H2c, H3c, and H4c.

## 5 Discussion

### 5.1 Meaningful experience as an important psychological starting point for the formation of happiness in smart hotels

The results showed that respondents generally recognized the convenience, technological value, and accommodation benefits associated with smart hotels. Specifically, meaningful experience exhibits a significant positive relationship with and displays predictive value for happiness.

This finding converges with prior investigations into eudaimonic tourism experiences (Filep and Deery, 2010), which suggest that cognitive appraisals of personal growth and life-enhancement function as core predictors of long-term subjective well-being. However, while traditional tourism literature typically ties eudaimonic quality to interpersonal, human-centric interactions or natural environments, the current study demonstrates that a eudaimonic state can also be predicted by technology-mediated lodging encounters. This presents an empirical contribution that stands in contrast to classical technology adoption studies in tourism (Kim et al., 2021), which predominantly view utilitarian convenience or transaction-specific satisfaction as the principal outcomes of technological interactions.

The distinction lies in the eudaimonic framing of meaningful experience: conventional satisfaction reflects whether a technology performs up to basic operational expectations, whereas a meaningful experience is associated with how consumers cognitively interpret these automated services as improving their well-being, supporting their psychological autonomy, or reflecting a preferred lifestyle. From the perspective of positive psychology, the psychological value of smart hotels is associated not only with operational usability but also with the cognitive process of meaning-making during service encounters. When technology-mediated services are evaluated as facilitating personal time-saving, comfort, and safety, they display a stronger positive association with subjective well-being.

### 5.2 Positive emotions as a key pathway linking meaningful experiences to happiness

The study found that smart hotel services are positively associated with positive emotions such as relaxation, enjoyment, novelty, and anticipation among consumers. The findings support a significant indirect pathway from meaningful experience to positive emotions, and subsequently to happiness.

This result aligns with the Broaden-and-Build Theory (Fredrickson, 2001), which proposes that positive emotional experiences temporarily expand an individual’s momentary thought-action repertoire, which in turn is associated with the accumulation of personal resources like happiness. This positive emotional pathway acts as a central psychological link translating immediate service value evaluations into broader, long-term assessments of well-being (Knobloch et al., 2017; Vada et al., 2020; Wang et al., 2021; Smith and Kawabata, 2025).

Interestingly, prior research on automated service interfaces often reports that technology-mediated encounters are associated with frustration, technology anxiety, or alienation due to impersonal machinery (Lu et al., 2020; Gursoy et al., 2019). The positive emotional pathway confirmed in this study presents divergent evidence. This discrepancy suggests that while raw technology exposure may induce friction, raising the cognitive appraisal of a smart service to a “meaningful” level (i.e., recognizing its eudaimonic value) relates positively to the elicitation of positive emotions, thereby mitigating potential technological friction. By positioning meaningful experience as a positive associate of emotional outcomes, this study contributes a useful boundary condition to the ongoing debate on automated emotional service design.

### 5.3 Perceived control and service trust as key psychological resources for positive experiences

The structural models reveal that both perceived control and service trust are positively associated with positive emotions and happiness, illustrating that they act as sequential and parallel mediation pathways. Crucially, the service trust pathway displays a stronger positive association with happiness compared to the perceived control pathway.

This positive role of control and trust as coping resources overlaps with recent research in hospitality automated systems (Sun et al., 2023; Tam et al., 2025). However, our finding that service trust is more closely related to happiness than perceived control diverges from typical findings in general software adoption studies focusing primarily on individual agency. In general technology adoption contexts, user evaluations are predominantly driven by agency and control. In the smart lodging context, however, the environment is a private living space where guests sleep, store personal items, and expect high safety. This unique context explains why service trust (representing privacy protection, system stability, and safety) exhibits a stronger positive relationship with customer happiness than the mere capacity to control the devices.

From the perspective of positive psychology, a sense of control and trust are important psychological resources. A sense of control is negatively related to technological anxiety, suggesting consumers believe they can handle issues in smart services; while trust is associated with psychological security, encouraging consumers to relax and enter smart service scenarios (Tam et al., 2025). Only when these two types of psychological resources are fully activated can the meaningful experience of smart hotels relate more strongly to positive emotions and happiness.

### 5.4 Service exposure differences suggest smart hotels still need to enhance sustained attractiveness

The distribution of service contact types mainly focused on self-check-in/check-out, facial recognition or smart door locks, smart guest room environment adjustment, online reservation and intelligent customer service, and intelligent voice control. Among them, self-check-in/check-out accounted for 20.55%, and facial recognition or smart locks for 17.48%, indicating that respondents are highly familiar with process-based procedures, identity verification, and contactless services. Personalized recommendation services account for 7.44%, robot delivery for 9.39%, and other services for 1.94%, indicating that the exposure rate for some deep intelligence services remains limited.

Recent online-review research shows that smart hotel competitiveness is increasingly reflected in consumers’ evaluations of technology-enabled service attributes, while service robot performance can function as an excitement factor in overall hotel satisfaction (Borghi and Mariani, 2024). The uneven exposure observed in the present sample may therefore indicate that some automated services have become familiar entry-level technologies, whereas more advanced robotic and personalized services remain less widely experienced.

### 5.5 Sample characteristics and the interpretive context of the findings

From the perspective of demographic characteristics, the sample showed a relatively balanced gender distribution. This balanced composition may reduce the likelihood that the interpretation of the findings is disproportionately associated with one gender group. Respondents aged 26–35 and 36–45 constituted over half (57.93%) of the sample. These age groups may have relatively greater exposure to travel, accommodation, and digital services, which may be associated with greater familiarity when evaluating smart hotel services.

Regarding educational attainment, 62.29% of the respondents held a bachelor’s degree or above. This educational profile, combined with the fact that all 618 participants in the finalized sample possessed direct stay experiences in smart hotels, suggests that the respondents had a solid understanding of both technology-mediated encounters and the survey items. This experiential and educational background provides a grounded, realistic basis for interpreting post-consumption evaluations (Yang et al., 2025).

Nevertheless, the composition of the sample may limit the extent to which the findings can be generalized to other consumer groups. Future research could include larger proportions of respondents with lower educational attainment, older consumers, infrequent hotel users, and consumers from cities of different administrative or development levels. In particular, respondents aged 56 years and above accounted for only 8.09% of the present sample. Future studies should therefore pay greater attention to how technological anxiety, perceived lack of control, and service-support needs may be associated with the smart hotel experiences of middle-aged and older consumers.

### 5.6 Theoretical contributions

This study makes three main theoretical contributions. First, it extends smart hotel research beyond standard frameworks of technology acceptance, service quality, customer satisfaction, and revisit intention by introducing meaningful experience and consumer happiness. Previous research has primarily modeled consumer evaluations focusing on utilitarian values and technology adoption intentions (Kim et al., 2021). By contrast, this study establishes that technology-mediated lodging experiences are positively associated with subjective happiness. This adds an empirical contribution by demonstrating that eudaimonic assessments (meaningful experience) represent a distinct cognitive predictor of customer well-being, which traditional transaction-specific satisfaction scales fail to capture.

Second, this study applies positive psychology and experience-centric theories to the highly digitalized hospitality domain through a multi-theory integration. Because technology-mediated lodging involves complex technological, cognitive, emotional, and eudaimonic dimensions, no single theoretical framework is sufficient to explain the overall psychological mechanism. By integrating these complementary perspectives, the framework clarifies the unique theoretical foundation supporting each structural path:

Self-Determination Theory (SDT) accounts for the direct link between meaningful experience (X) and happiness (Y), explaining how automated service encounters that satisfy intrinsic psychological needs (such as autonomy and competence) relate to well-being.

The Broaden-and-Build Theory explains the emotional mediation pathway (PE → Y), showing how transient positive emotions built during digital interactions are associated with broader, long-term evaluations of happiness.

Cognitive Appraisal Theory models the pathways linking meaningful experience (X) to perceived control (PC) and service trust (ST), clarifying how cognitive evaluations of service meaning relate to the mobilization of coping resources.

Meanwhile, while Service-Dominant (S-D) Logic, Experience Economy Theory, and Customer Experience Theory explain how meaningful experiences (X) are co-created at technological touchpoints rather than passively received, Smart Tourism Ecosystem Theory provides the overarching rationale showing how these concurrent pathways successfully coexist within digital service networks.

Third, this study offers methodological and conceptual clarity for hospitality literature by aligning sample characteristics with experiential evaluations. Unlike studies that frequently combine aware-only consumers with experienced users (which risks confounding expected utility with experienced utility), this study bases all structural estimations exclusively on a verified sample of consumers with actual smart hotel stays (*N* = 618). This sample design eliminates potential confounding effects, providing a cleaner and more realistic empirical baseline for understanding how automated service experiences relate to customer well-being.

### 5.7 Practical implications

#### 5.7.1 Implications for smart hotel managers and technology designers

To translate the empirical findings into actionable hospitality practices, hotel managers and system designers should align system optimizations with the two core mediating pathways verified in the model: perceived control (PC) and service trust (ST).

Supporting Perceived Control (PC): Because perceived control is positively associated with positive emotions and customer happiness, system design should reduce operational complexity. Designers should implement simplified, layered interaction structures and one-step menu pathways on hotel applications and self-service kiosks to lower the cognitive burden. Additionally, smart guest rooms must feature a physical manual-override option, allowing guests to toggle easily between automated AI settings and manual operations, which supports their subjective agency.

Supporting Service Trust (ST): Since service trust is the stronger predictor of happiness in the private accommodation environment, hoteliers must guarantee system reliability. Digital interfaces should display real-time feedback messages during system processing and feature prominent, simplified privacy statements regarding data protection. Most importantly, even in highly automated environments, designers should embed a direct human-on-call fallback channel into the smart hotel app, ensuring that guests can obtain immediate human assistance if a tech failure occurs, thereby preventing a deficit in service trust.

#### 5.7.2 Implications for tourism policymakers

As a broader managerial consideration, industry regulators should establish standardized frameworks to support consumer interests in the digitized lodging market:

Securing Service Trust (ST): Policymakers should consider implementing unified industry standards for customer data protection in smart hospitality, establishing clear boundaries for biometric verification (such as facial recognition locks) and regulatory requirements for guest record storage.

Securing Perceived Control (PC): Regulatory agencies are encouraged to design basic quality evaluation benchmarks for smart lodging properties. These benchmarks should assess system ease of use, manual backup availability, and safety designs, helping to ensure that digital lodging services remain accessible and user-friendly for older or technology-anxious consumer segments.

## 6 Conclusion and limitations

### 6.1 Research conclusion

Based on 618 valid questionnaires collected from consumers with actual smart hotel stay experiences, this study examines the associations among meaningful experience, positive emotions, perceived control, service trust, and happiness. The results indicate that meaningful experience is positively associated with consumer happiness and that positive emotions statistically mediate this relationship. Perceived control and service trust are also indirectly associated with happiness through positive emotions.

The findings suggest that the value of smart hotels extends beyond technological efficiency to include the extent to which technology-enabled services are perceived as meaningful, controllable, trustworthy, and emotionally positive. Technological efficiency may provide the functional basis of smart hotel services, whereas meaning construction, positive emotions, perceived control, and service trust may contribute to explaining their associations with consumer happiness. From this perspective, the transition from “intelligent accommodation” to “happiness-oriented experiences” may depend on whether technology is aligned with consumers’ positive experiential and psychological needs.

### 6.2 Limitations and future research

This study has several limitations that suggest directions for future research.

First, the use of self-reported data may introduce common method bias and subjective perception bias, although statistical design and post-hoc tests were applied to mitigate these issues. Future studies should consider using behavioral, physiological, or multi-source data to evaluate these associations.

Second, the cross-sectional design prevents the establishment of temporal ordering and definitive causal relationships among the variables. The pathways examined in this model should be interpreted as predictive and statistical associations rather than causal effects. Future studies could employ longitudinal, time-lagged, or experimental designs to further clarify the directionality and psychological processes underlying these associations.

Third, the convenience sampling approach may limit the generalizability of the findings despite stratified controls. Future studies should adopt probability sampling methods or larger, nationally representative samples.

Fourth, the single-country context may restrict cross-cultural applicability, as perceptions of smart hotel services likely vary across cultures and institutions. Multi-country comparisons are needed.

Fifth, differences in smart hotel market maturity may influence user trust and perceived control, suggesting the need to consider contextual development stages in future analyses.

Finally, future research should examine moderating effects, such as age, technology readiness, and privacy concerns, to better explain heterogeneous consumer well-being outcomes in automated hospitality settings.

Overall, these limitations highlight boundary conditions of the study and provide directions for further research on smart hospitality and positive psychology mechanisms.

## Data Availability

The original contributions presented in the study are included in the article/Supplementary material, further inquiries can be directed to the corresponding author/s.
