# Supplementary material for: Meaningful experience, positive emotions, and happiness in smart hotel contexts: a study on psychological mechanisms based on positive psychology
Source: Front Psychol. 2026 Jul 29;17:1897945. doi: 10.3389/fpsyg.2026.1897945 (PMC13461548; doi:10.3389/fpsyg.2026.1897945)
Supplement: Supplementary file 1 [file Data_Sheet_1.ZIP › Questionnaire.docx]

**Survey on Meaning Experience, Positive Emotions, and Happiness in Smart Hotel Contexts**

**Dear Interviewee:**

Hello! This questionnaire aims to understand consumers' meaningful experiences, positive emotions, and happiness during their stay or experience at smart hotels. The questionnaire is for academic research only; all information will be kept strictly confidential and will not involve personal identification. Please answer based on your own true feelings; there is no right or wrong answer.

**The survey targets adults aged 18 and above. If you are under 18 years old, please do not complete this questionnaire.**

Please choose the one that best fits your actual situation.

Scale description: 1 = Strongly disagree; 2 = Disagree; 3 = Average; 4 = Agree; 5 = Strongly agree.

**Voluntary Participation and Right to Withdraw**

Your participation is completely voluntary. You have the right to withdraw from the study at any time for any reason without any adverse consequences. If you feel uncomfortable or do not wish to continue during the completion process, you may simply close the page.

**Consent Statement**

I have carefully read and understood all the above content and am clearly aware of my rights and obligations. I voluntarily participate in this study and agree that the research team may collect and use the anonymous data I provide for academic research purposes.

□ I agree to participate in this study □ I do not agree to participate in this study

# Part One: Basic Information

**1. Your gender:**

A. Male

B. Female

**2. Your age:**

A. 18–25

B. 26–35

C. 36–45

D. 46–55

E. 56 years old and above

**3. Your level of education:**

A. High school and below

B. Junior college

C. Bachelor's degree

D. Master's degree or above

**4. Your Career:**

A. Students

B. Corporate employees

C. Staff of government agencies/public institutions

D. Enterprise managers

E. Self-employed individuals/freelancers

F. Others

**5. Your annual income level:**

A. Below 30，000 yuan

B. 30，000-60，000 yuan

C. 60，000-100，000 yuan

D. 100，000-150，000 yuan

E. 150，000 yuan or more

**6. Have you ever stayed or experienced a smart hotel?**

A. Yes.

B. No, but I do understand the related services

**7. How often have you stayed at Smart Hotels in the past year?**

A. Once or fewer

B. 2–3 times

C. 4–6 times

D. 7–10 times

E. 11 times or more

**8. The smart hotel services you have encountered**

A. Self check-in/check-out

B. Facial recognition or smart door locks

C. Intelligent voice control

D. Robot delivery

E. Smart guest room environment adjustment

F. Personalized recommendation services

G. Online Appointment and Intelligent Customer Service

H. Others

## Part Two: Smart Hotel Meaning Experience Scale

Please rate based on your actual experience in the smart hotel setting.

| **Number** | **Topic** | **1** | **2** | **3** | **4** | **5** |
| --- | --- | --- | --- | --- | --- | --- |
| X1 | The services at Smart Hotel made my stay feel even more worthwhile. |  |  |  |  |  |
| X2 | The intelligent services of smart hotels made me feel the significance of technology improving life. |  |  |  |  |  |
| X3 | In smart hotels, I can experience a more convenient and efficient lifestyle. |  |  |  |  |  |
| X4 | The experience of smart hotels has inspired me to imagine a positive way of life in the future. |  |  |  |  |  |
| X5 | Smart hotels make me feel that my spending choices better meet the needs of modern life. |  |  |  |  |  |
| X6 | Smart hotel services not only meet accommodation needs but also bring new experiential value. |  |  |  |  |  |
| X7 | Smart hotels make my journey or stay feel more meaningful. |  |  |  |  |  |

## Part Three: Positive Emotions Scale

Please evaluate based on your emotional feelings during your smart hotel experience.

| **Number** | **Topic** | **1** | **2** | **3** | **4** | **5** |
| --- | --- | --- | --- | --- | --- | --- |
| PE1 | The services of Smart Hotel made me feel very happy. |  |  |  |  |  |
| PE2 | I feel at ease using smart hotel services. |  |  |  |  |  |
| PE3 | The intelligent experience of smart hotels is novel to me. |  |  |  |  |  |
| PE4 | The convenience of smart hotels gives me positive feelings. |  |  |  |  |  |
| PE5 | When I stayed at the Smart Hotel, I was in a good overall mood. |  |  |  |  |  |
| PE6 | The interactive experience in the smart hotel made it interesting to me. |  |  |  |  |  |
| PE7 | The services of smart hotels made me look forward to my stay. |  |  |  |  |  |

## Part Four: Perceived Control Scale

Please rate based on your sense of control during the use of smart hotel services.

| **Number** | **Topic** | **1** | **2** | **3** | **4** | **5** |
| --- | --- | --- | --- | --- | --- | --- |
| PC1 | I can easily understand how various smart services in smart hotels are used. |  |  |  |  |  |
| PC2 | When using smart hotel services, I find the operation process quite clear. |  |  |  |  |  |
| PC3 | I can choose whether to use the hotel's smart services. |  |  |  |  |  |
| PC4 | When problems arise, I believe I can find a solution. |  |  |  |  |  |
| PC5 | The service process of a smart hotel feels controllable to me. |  |  |  |  |  |
| PC6 | The smart hotel doesn't cause me any obvious discomfort or a sense of loss of control. |  |  |  |  |  |

## Part Five: Service Trust Scale

Please rate based on your trust in the smart hotel system, security, and service reliability.

| **Number** | **Topic** | **1** | **2** | **3** | **4** | **5** |
| --- | --- | --- | --- | --- | --- | --- |
| ST1 | I believe the overall smart systems of smart hotels are reliable. |  |  |  |  |  |
| ST2 | I believe the smart hotel can protect my personal information. |  |  |  |  |  |
| ST3 | I believe the smart services of smart hotels can operate stably. |  |  |  |  |  |
| ST4 | I believe smart hotels can handle service issues promptly. |  |  |  |  |  |
| ST5 | The smart devices in smart hotels make me feel safe. |  |  |  |  |  |
| ST6 | I am willing to trust the personalized service recommendations offered by smart hotels. |  |  |  |  |  |

## Part Six: Well-being Scale

Please choose based on how the smart hotel experience affects your overall impression and quality of life evaluation.

| **Number** | **Topic** | **1** | **2** | **3** | **4** | **5** |
| --- | --- | --- | --- | --- | --- | --- |
| Y1 | The smart hotel experience has improved my satisfaction with my accommodation life. |  |  |  |  |  |
| Y2 | Smart hotels make life feel more convenient for me. |  |  |  |  |  |
| Y3 | The smart hotel experience has made me feel my quality of life has improved. |  |  |  |  |  |
| Y4 | The comfort brought by smart hotels has enhanced my sense of happiness. |  |  |  |  |  |
| Y5 | Smart hotels let me experience the wonderful life brought by technological services. |  |  |  |  |  |
| Y6 | Overall, the smart hotel experience made me feel satisfied and happy. |  |  |  |  |  |
| Y7 | I believe the smart hotel experience can improve my travel or accommodation experience. |  |  |  |  |  |
